# From antenatal preparedness to enacted agency during labor: Integrated findings from a woman-centered intervention on mobility

**DOI:** 10.18332/ejm/223963

**Published:** 2026-08-31

**Authors:** Marlene Isabel Lopes, Alexandrina Cardoso

**Affiliations:** 1 University of Coimbra, Health Sciences Research Unit: Nursing (UICISA: E), Nursing School of University of Coimbra, Coimbra, Portugal; 2 Faculty of Health Sciences and Nursing, Universidade Católica Portuguesa, Centre for Interdisciplinary Research in Health, Porto, Portugal; 3 Nursing School, University of Porto, RISE-Health, Porto, Portugal

**Keywords:** antenatal education, agency, decision-making, labor mobility, childbirth experience

## Abstract

**INTRODUCTION:**

Freedom of movement and upright positions during labor are recommended in maternity care, yet organizational routines may constrain women’s mobility, autonomy, and shared decision-making. This study examined how a woman-centered antenatal intervention on mobility during labor operated and under which conditions its effects were enabled or constrained.

**METHODS:**

A convergent mixed-methods study was conducted in community-based childbirth preparation programs in Portugal. Quantitative data were collected from low-risk nulliparous women at baseline (n=56), post-intervention (n=38), and postpartum (n=30), assessing emancipated decision-making, decision satisfaction, childbirth self-efficacy, birth beliefs, and childbirth experience. Semi-structured interviews were conducted with eight women and four nurse-midwives. Data were analyzed separately and integrated through joint display analysis and meta-inferences.

**RESULTS:**

Satisfaction with decision-making improved significantly (3.66 vs 3.70, p<0.001), natural birth beliefs increased (4.14 vs 4.41, p<0.001), and medical birth beliefs decreased (3.74 vs 3.51, p=0.032). No significant antenatal changes were found in emancipated decision-making or childbirth self-efficacy. Women who remained mobile during labor reported higher overall childbirth experience (p=0.005), participation (p=0.011), own performance (p=0.011), and professional support (p=0.015). Qualitative findings showed acceptability and feasibility, highlighting experiential learning, bodily awareness, reflective preparation, and nurse-midwife facilitation as key mechanisms. Integrated findings suggested that antenatal measures primarily captured preparedness for action, whereas agency became more visible when women enacted mobility during labor.

**CONCLUSIONS:**

The intervention may strengthen women’s preparedness to act regarding mobility during labor, but enacted agency appears to depend on intrapartum relational and organizational conditions.

## INTRODUCTION

A positive childbirth experience is a central goal of high-quality maternity care and an important outcome for women and families^1^. Beyond clinical safety, women’s experiences of labor and birth are shaped by whether they feel involved, respected, and able to participate actively in decisions about their care. Perceived control during labor has been consistently associated with greater satisfaction, better psychological adjustment, and more positive childbirth experiences^2,3^.

Among the practices that can support women’s participation and sense of control during labor, freedom of movement and upright positions are particularly relevant. These practices are recommended in international guidance and supported by evidence showing benefits such as shorter labor, fewer interventions, and improved maternal and neonatal outcomes^1,4,5^. However, despite this evidence, mobility during labor remains constrained in some hospital settings. Institutional routines, continuous monitoring practices, and dominant biomedical models of care may favor recumbent or semi-recumbent positions, sometimes irrespective of women’s preferences or comfort^6-9^. These constraints may limit women’s opportunities to act on their preferences and may weaken their sense of agency during labor^10^.

Antenatal education is one possible route for supporting women’s active participation in labor. At its best, it can do more than convey information: it can help women interpret labor as a process in which they can act, make choices, and mobilize internal and external resources. Antenatal preparation may strengthen confidence, coping, and decision-making, and may contribute to more positive experiences of childbirth^11,12^. Yet, preparing women to remain mobile and make decisions in labor involves more than just increasing knowledge. It also requires opportunities to develop bodily awareness, clarify preferences, and anticipate how to respond in care contexts that may or may not support autonomy and shared decision-making^13,14^.

Although woman-centered care has received increasing attention, relatively few antenatal interventions have been explicitly designed to support women’s decision-making and agency in relation to mobility during labor. In addition, much of the available literature has focused on measurable outcomes, offering more limited insight into how such interventions work, which mechanisms appear to matter, and under what contextual conditions their effects are enabled or constrained^15,16^. In particular, little is known about how antenatal preparation translates into enacted agency during labor.

To address this gap, a woman-centered antenatal intervention was developed and integrated into community-based childbirth preparation programs (CPPs) led by nurse-midwives in Portugal. Previous publications have reported on the development, feasibility, and strand-specific quantitative and qualitative findings of the intervention^14,17-19^. The present paper reports the meta-inferences generated by bringing both strands together. Using a convergent mixed-methods design, this study aimed to examine how the intervention operated across the antenatal and intrapartum periods, through which mechanisms it appeared to support women’s decision-making and agency regarding mobility during labor, and under which relational and organizational conditions these effects were facilitated or constrained.

## METHODS

### Study design

This study used a convergent mixed-methods design to examine the preliminary effects of a woman-centered antenatal intervention on mobility during labor, alongside its acceptability, feasibility, and perceived mechanisms of impact. The study was conducted within the Medical Research Council (MRC) framework for the development and evaluation of complex interventions^20^.

The quantitative and qualitative strands were implemented during the feasibility phase, analyzed separately, and integrated after the completion of strand-specific analyses. A convergent design was chosen because the study sought not only to explore whether the intervention was associated with measurable changes, but also to understand how it functioned, through which mechanisms it appeared to operate, and under which relational and organizational conditions its effects were enabled or constrained. Integration was therefore used to generate meta-inferences that could not be derived from either strand alone, with particular attention to the relationship between antenatal preparation and the enactment of agency during labor^21^.

### Intervention

The intervention, ‘I choose to move during my labor’, was a woman-centered antenatal midwifery intervention integrated into existing community-based CPPs delivered by nurse-midwives in Portuguese primary care settings. It aimed to strengthen women’s knowledge, confidence, reflective decision-making, communication, and practical preparedness regarding mobility and upright positions during labor. The intervention comprised two sequential face-to-face group sessions of 2.5 hours each, delivered to groups of up to eight women and their partners or significant others. To promote consistency, facilitators used a structured intervention guide, and participants received illustrated leaflets with labor positions, movements, and suggestions for partner support. The intervention was organized around four interrelated components: group-based face-to-face delivery, experiential learning, decision-making support, and a safe environment fostered through positive communication. It was theoretically informed by the theory of emancipated decision-making in women’s health care^22^ and Bandura’s self-efficacy theory^23^.

The first session, ‘Moving during labor: my superpower!’, focused on experiential learning and bodily awareness. Women practiced movements and upright positions intended to support comfort, labor progress, and active participation, including adaptations for use in bed or in more restricted clinical contexts. Activities included observation, guided practice, use of resources such as the birth ball or peanut ball, group reflection, and relaxation exercises combining conscious breathing, mindfulness, positive affirmations, and connection with the baby. This session aimed to help women recognize their bodily resources and rehearse strategies adaptable to the circumstances of labor.

The second session, ‘I choose to move during my labor’, focused on decision-making training and support. Women explored hypothetical labor scenarios involving situations that could influence mobility, including hospital admission, transfer to the birth room, clinical instructions, monitoring, or epidural analgesia. Guided reflection was supported by the BRAIN model (Benefits, Risks, Alternatives, Intuition, and Nothing) to help women consider options, clarify preferences, anticipate barriers, and practice communication with health professionals.

The intervention explicitly combined practical rehearsal of mobility and upright positions with structured decision-support and communication strategies. It sought to prepare women to interpret labor situations, express preferences, and make mobility-related decisions where clinical and organizational conditions allowed.

### Quantitative strand

*Participants and data collection*

The quantitative strand used a pre-experimental pretest-posttest design with postpartum follow-up. Participants were low-risk nulliparous women attending antenatal CPPs in Community Care Units (CCUs) in Portugal. Data were collected at three time points: baseline before the intervention (T1), post-intervention during pregnancy (T2), and postpartum follow-up (T3). Validated Portuguese versions of the following instruments were administered: the Birth Beliefs Scale (BBS-pt)^24^, the Revised Emancipated Decision-Making Scale (EDMr-pt)^25^, the Satisfaction With Decision Scale (SWD-pt)^26^, and the Childbirth Self-Efficacy Inventory (CBSEI-pt)^27^. At postpartum follow-up, the Childbirth Experience Questionnaire (CEQ-pt)^28^ was completed, and additional clinical and obstetric data related to labor, mobility, and birth outcomes were collected^18^.

*Data analysis*

Descriptive statistics were used to characterize the sample. Changes between T1 and T2 were examined using paired-sample or non-parametric tests, according to data distribution. Exploratory correlational and regression analyses were conducted to examine associations between post-intervention variables and childbirth experience outcomes at T3. Analyses were performed using IBM SPSS Statistics, version 29.0 (IBM Corp., Armonk, NY, USA), and statistical significance was set at p<0.05.

### Qualitative strand

*Participants and data collection*

The qualitative strand comprised a retrospective descriptive study embedded within the feasibility study. Semi-structured interviews were conducted with two groups of participants: women who had received the intervention and subsequently given birth, and nurse-midwives who had implemented the intervention.

Women were interviewed approximately one month postpartum, allowing them to reflect on the intervention in light of their labor and birth experiences. Nurse-midwives were interviewed after the implementation period had concluded. The interviews explored perceived acceptability and feasibility^19^.

*Data analysis*

Interview data were analyzed using thematic analysis informed by the Theoretical Framework of Acceptability^29^, and by the MRC Process Evaluation Framework^30^. Coding and theme development were iterative and reflexive, with ongoing movement between data, codes, and developing interpretations to support analytic depth and transparency.

### Integration of quantitative and qualitative data

Integration occurred after completion of the strand-specific analyses and was undertaken at the interpretive level. Quantitative and qualitative findings were brought together through joint display analysis to identify areas of convergence, complementarity, and divergence, and to examine how qualitative insights helped explain, extend, or contextualize the quantitative results^21,31^.

The two strands were given complementary priority. Quantitative findings were used to identify measurable changes and associations across the antenatal and postpartum periods, whereas qualitative findings were used to clarify how these patterns were experienced, how participants interpreted the intervention, and under what conditions preparation was or was not translated into action during labor^31^. Through this process, meta-inferences were developed and synthesized into an explanatory model of how the intervention appeared to support women’s preparedness for mobility-related decision-making and how enacted agency depended on intrapartum relational and organizational conditions.

## RESULTS

### Quantitative findings

Fifty-six women completed the baseline assessment (T1), 38 completed the post-intervention assessment during pregnancy (T2), and 30 provided valid postpartum follow-up data (T3). Detailed participant flow and attrition have been reported elsewhere^18^. Among women who completed both antenatal assessments, the mean age was 31.97 years (SD=5.03), and most were Portuguese, employed, living with a partner, and had completed higher education.

Comparison of baseline and post-intervention scores showed significant improvements in satisfaction with decision-making and in birth beliefs, with greater endorsement of birth as a physiological process and lower endorsement of birth as a medical and risky event. No statistically significant antenatal pre-post changes were observed in emancipated decision-making or childbirth self-efficacy (Table 1).

**Table 1:** Preliminary pre–post changes in decision-making, satisfaction with decision, childbirth self-efficacy, and birth beliefs among low-risk nulliparous women participating in a community-based antenatal mobility intervention in Portugal (N=38)

*Instruments*	*T1 Mean (SD)*	*T2 Mean (SD)*	*ΔMean*	*Test t (df)*	*p*	*Effect size*
Emancipated Decision-Making (EDMr-pt)	3.66 (0.34)	3.70 (0.32)	0.05	t(37)= -0.637	0.528	d=0.10
Satisfaction with Decision (SWD-pt)	23.05 (3.00)	25.37 (2.45)	2.32	t(37)= -3.675	<0.001	d=0.60
Childbirth Self-Efficacy (CBSEI-pt)	7.61 (1.58)	7.76 (1.43)	0.15	t(37)= -0.854	0.399	d=0.14
Birth Beliefs – Natural (BBS-pt)	4.14 (0.44)	4.41 (0.40)	0.27	Z=3.764	<0.001	R=0.61
Birth Beliefs – Medical (BBS-pt)	3.74 (0.65)	3.51 (0.70)	-0.24	t(37)=2.229	0.032	d=0.36

ΔMean=T2-T1. Effect sizes interpreted according to Cohen’s thresholds: small=0.10; medium=0.30; large=0.50.

At postpartum follow-up, most women reported remaining mobile during the dilation phase, although more than half reported reduced mobility after transfer to the birth room. Women who remained mobile during labor reported more positive childbirth experiences, particularly in relation to participation, their own performance, and professional support. Mobility was also associated with mode of birth, although this finding should be interpreted cautiously given the very small number of women in the non-mobile group. No statistically significant differences were observed in labor duration or epidural use, although a trend towards later epidural administration was identified among women who remained mobile.

Exploratory correlational analyses between post-intervention variables and childbirth experience showed no significant associations between emancipated decision-making, satisfaction with decision-making, childbirth self-efficacy, or natural birth beliefs and the overall CEQ-pt score. However, natural birth beliefs were positively associated with the CEQ-pt participation subscale (ρ=0.373, p=0.042). Exploratory regression analysis showed that the model including emancipated decision-making, satisfaction with decision-making, childbirth self-efficacy, and natural birth beliefs did not significantly predict overall childbirth experience [F(4, 25)=0.865, p=0.498, R²=0.122, adjusted R²= -0.019]. None of the individual predictors reached statistical significance. Regression models for the CEQ-pt subscales were also non-significant, although natural birth beliefs approached significance as a predictor of participation (β=0.447, p=0.062).

Greater satisfaction with the intervention was positively associated with overall childbirth experience (r=0.444, p=0.014), own performance (r=0.469, p=0.009), and professional support (ρ=0.376, p=0.041). No significant correlations were found with the participation or own threshold subscales.

### Qualitative findings

Semi-structured interviews were conducted with eight women and four nurse-midwives. Overall, both groups described the intervention as acceptable and feasible. Women emphasized the value of the group format, experiential learning, reflection on realistic scenarios, and printed materials that could be recalled during labor. These elements appeared to support bodily awareness, confidence, and preparation to express preferences and use movement strategies.

Nurse-midwives highlighted the intervention’s structured and experiential character and described it as contributing to a more woman-centered educational approach. Women’s postpartum accounts suggested that the intervention helped them interpret bodily cues, remain active, and make context-sensitive decisions during labor. At the same time, both women and nurse-midwives identified institutional routines, clinical instructions, limited space, and organizational constraints as factors that could restrict the application of antenatally rehearsed strategies^19^.

### Integrated findings

Integration of the quantitative and qualitative findings provided a more comprehensive understanding of how the intervention appeared to operate, what kinds of changes it supported, and under which conditions those changes were or were not translated into action. Table 2 presents the joint display used to compare and integrate findings across key analytical dimensions.

**Table 2:** Joint display integrating quantitative and qualitative findings from a convergent mixed methods feasibility study of a woman-centered antenatal intervention on mobility during labor

*Analytical dimension*	*Quantitative findings (women)*	*Qualitative findings (women/nurse-midwives)*	*Integrated inference*
**1. Decision-making**	Satisfaction with decision increased: t(37)= -3.675, p<0.001, d=0.60. No significant change in EDMr-pt: p=0.528, d=0.10.	Women described feeling more able to consider options, express preferences, and anticipate ‘what if’ situations. Nurse-midwives viewed decision-focused work as innovative, although one expressed concern that it might heighten anxiety for some women.	The intervention showed clearer antenatal effects on decision satisfaction than on emancipated decision-making or self-efficacy as measured during pregnancy. Qualitative findings suggested that agency and self-efficacy became more visible when women encountered real labor situations. Reflective decision support was valued by participants, but it also raised some implementation challenges, particularly regarding professionals’ comfort with this depth of reflection.
**2. Birth beliefs**	Natural birth beliefs increased: Z=3.764, p<0.001, r=0.61. Medical birth beliefs decreased: t(37)=2.229, p=0.032, d=0.36.	Women described reframing birth through embodied learning and positive language, with greater trust in the body. Midwives emphasized the value of visual and experiential tools in making labor physiology understandable.	A key mechanism of impact was cognitive and embodied reframing of childbirth as a physiological process, which appeared to support readiness for active participation.
**3. Self-efficacy and agency**	No statistically significant change in CBSEI-pt from T1 to T2: p=0.399, d=0.14.	Postpartum accounts described enacted confidence, self-regulation, use of movement, negotiation with professionals, and adaptive coping. Some women also reported frustration when strategies could not be used.	In this sample of nulliparous women, antenatal self-efficacy scores may have reflected anticipated rather than enacted confidence. Qualitative accounts indicated that self-efficacy and agency became more visible when women encountered real labor situations and attempted to apply the strategies rehearsed antenatally.
**4. Mobility during labor**	90% reported mobility during the dilation phase; >50% reported reduced mobility after transfer to the birth room. Mobility was associated with mode of birth: χ²(2)=6.296, p=0.043 (interpret cautiously due to very small non-mobile group).	Women described mobility as central to comfort, control, and confidence. Partners supported movement, and antenatally rehearsed strategies were used during labor. Mobility was often constrained after transfer to the birth room.	Mobility functioned as a behavioral bridge between antenatal preparation and labor experience, but its enactment depended on the care environment.
**5. Childbirth experience**	Mobility was associated with higher CEQ total (p=0.005) and higher own performance (p=0.011), participation (p=0.011), and professional support (p=0.015). Satisfaction with the intervention was positively associated with CEQ-pt total (r=0.444, p=0.014), own performance (r=0.469, p=0.009), and professional support (ρ=0.376, p=0.041). Exploratory regression models predicting CEQ-pt total and subscale scores were not statistically significant.	Women linked movement to stronger participation, perceived competence, bodily control, and more supportive professional interactions. Frustration emerged when preferences could not be enacted.	Positive childbirth experiences were concentrated in agency-related dimensions, suggesting that antenatal preparation was most relevant when women were able to enact movement, participation, and interaction with professionals during labor.
**6. Contextual conditions**	More than half of women reported reduced mobility after transfer to the birth room.	Women described intrapartum routines, clinical instructions, and limited flexibility in hospital care as constraints on applying strategies rehearsed antenatally. Nurse-midwives, in turn, identified barriers related mainly to intervention delivery, including limited space, large group sizes, and organizational constraints.	Contextual conditions operated at two levels: they influenced how the intervention could be delivered antenatally and how far women were able to enact preparation during labor. In particular, the translation of preparation into action depended on supportive intrapartum relational and organizational conditions.
**7. Feasibility**	High satisfaction with the intervention (mean 9.73/10)	Facilitators included low cost, easy integration, high participant engagement, and professional learning. Constraints included space, large groups, and variable comfort with decision-focused facilitation.	The intervention appeared feasible and acceptable, but implementation quality depended on practical conditions and professional alignment with participatory approaches.

Taken together, the integrated findings supported four main inferences regarding how the intervention operated and under which conditions its effects were realized.

### Inference 1: The intervention primarily strengthened antenatal preparedness rather than measurable antenatal agency

Quantitative findings showed clear improvements in satisfaction with decision-making and in beliefs aligned with birth as a physiological process, whereas no significant pre-post changes were observed in emancipated decision-making or childbirth self-efficacy. Qualitative findings help explain this pattern. Women described increased confidence, clearer reflection on options, greater bodily awareness, and a stronger sense of readiness to participate in decisions. Together, these findings suggest that the intervention more readily supported antenatal preparedness for action than deeper or more context-dependent forms of agency measurable during pregnancy.

### Inference 2: Agency and self-efficacy became more visible when enacted during labor

The lack of significant quantitative change in antenatal self-efficacy and emancipated decision-making did not align straightforwardly with women’s postpartum narratives, which frequently described active coping, negotiation, movement, and context-sensitive decision-making during labor. Integration therefore suggested a temporal distinction between anticipated agency, assessed during pregnancy, and experienced or enacted agency, which became visible during labor itself. In this sense, labor functioned as the context in which women’s preparedness could be translated into action, but also as the context in which the limits of that preparedness became evident.

### Inference 3: Mobility acted as a behavioral bridge between antenatal preparation and childbirth experience

Quantitative findings showed that women who remained mobile during labor reported more positive childbirth experiences, particularly in the domains of participation, own performance, and professional support. Qualitative accounts reinforced this pattern by describing mobility as central to comfort, control, confidence, and alignment between bodily sensations, personal preferences, and interaction with care providers. Taken together, these findings suggest that mobility functioned as a behavioral bridge between antenatal preparation and experiential outcomes during childbirth.

### Inference 4: Context shaped both how the intervention was delivered and how preparation was enacted during labor

Both strands highlighted the importance of context, although in different ways. Quantitative findings showed that more than half of the women reported reduced mobility after transfer to the birth room. In the interviews, women described intrapartum routines, clinical instructions, and limited flexibility in hospital care as constraints on applying strategies rehearsed antenatally. Nurse-midwives, in turn, referred mainly to implementation-related barriers, including limited space, large group sizes, and some difficulty in facilitating the decision-focused component. Together, these findings indicate that contextual influences operated at two levels: first, in shaping how the intervention could be delivered antenatally, and second, in shaping whether women could translate preparation into enacted agency during labor.

**Figure 1:**
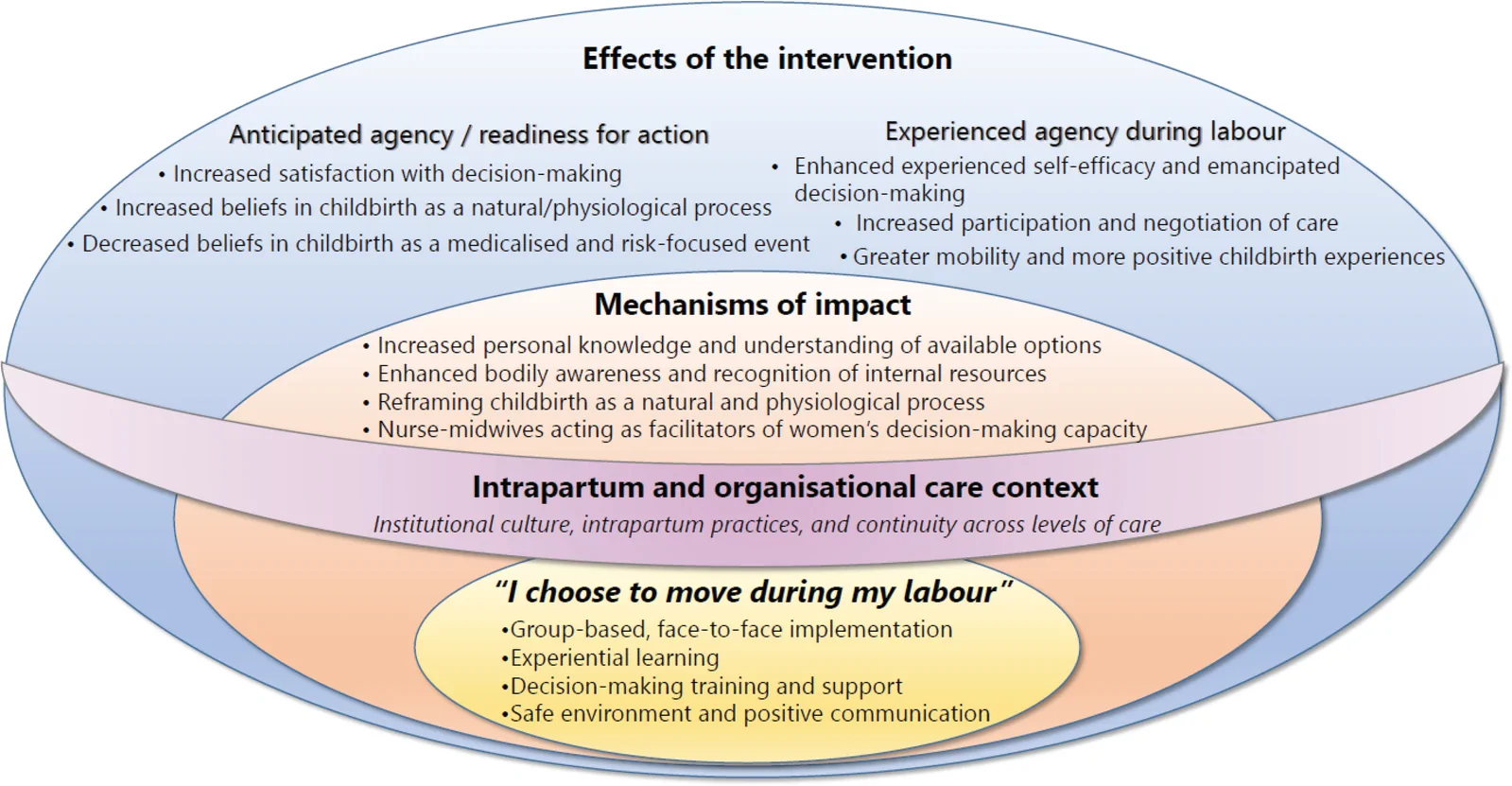
Integrated model of the woman-centered antenatal intervention, illustrating its core components, mechanisms of impact, and effects across pregnancy and childbirth

Overall, the integrated analysis suggests that the intervention operated through cognitive, embodied, and relational mechanisms. Its most visible antenatal effects involved interpretive and decisional preparation, while its fuller effects appeared during labor when women were able to translate preparation into movement, participation, and interaction within supportive care environments. Figure 1 presents the explanatory model developed through mixed methods integration. The model synthesizes how the intervention appeared to function across the antenatal and intrapartum periods, the mechanisms through which it supported change, and the contextual conditions that influenced whether its effects could be enacted in practice.

At the center of the model is the intervention ‘I choose to move during my labor’, delivered in a group-based and experiential format to support women’s decision-making in relation to mobility and upright positions during labor. Through its core components, the intervention appeared to activate cognitive, embodied, and relational mechanisms, including increased knowledge of available options, greater bodily awareness, reframing of birth as a physiological process, and supportive facilitation by nurse-midwives. These mechanisms appeared to generate different kinds of effects across pregnancy and childbirth. During pregnancy, the intervention was associated primarily with preparedness for action, expressed through increased satisfaction with decision-making and changes in birth beliefs. During labor, agency became more visible as women attempted to apply movement strategies, negotiate care, and participate actively in their childbirth experience. However, the model also highlights that this translation from preparation to action was contingent on context. Intrapartum routines, institutional culture, and continuity between antenatal and hospital care could either enable or constrain the enactment of agency.

## DISCUSSION

This mixed-methods study integrated quantitative and qualitative findings to examine how a woman-centered antenatal intervention on mobility during labor appeared to operate, what kinds of changes it supported, and under which conditions those changes could be enacted. Overall, the integrated findings suggest that the intervention acted through cognitive, embodied, and relational mechanisms. Its most visible antenatal effects were seen in women’s decisional preparedness and childbirth beliefs, whereas agency became more clearly visible when women attempted to enact mobility and decision-making during labor. Importantly, this enactment was shaped by the relational and organizational conditions of intrapartum care.

A central finding of this study is that the intervention appeared to do more than provide information. Rather than functioning as a conventional educational input, it seemed to support women’s preparedness for action by combining knowledge, experiential learning, bodily awareness, and facilitated reflection. This interpretation is consistent with key dimensions of Emancipated Decision-Making theory, particularly the importance of personal knowledge, relational support, and conditions that enable women to act on their values and preferences^22^.

Quantitatively, this pattern was reflected in significant improvements in satisfaction with decision-making and in beliefs more strongly aligned with birth as a physiological process. Qualitative accounts help explain how these changes may have occurred. Women described that embodied experimentation with movement and positions, reflection on realistic scenarios, and the use of positive and symbolic language helped them clarify preferences, anticipate challenges, and feel more confident about their options. Nurse-midwives also described the intervention as more experiential, more structured, and more aligned with woman-centered educational practice.

These findings support the view that antenatal decision-support interventions may be more meaningful when they engage cognitive, embodied, and relational processes rather than relying on information transfer alone^14,16^. In this study, satisfaction with decision-making appeared to function as an early and sensitive indicator of impact, capturing women’s sense of clarity, confidence, and alignment between values and intended action.

The most important integrative contribution of this study lies in the distinction between preparedness during pregnancy and agency enacted during labor. No statistically significant antenatal pre-post changes were observed in emancipated decision-making or childbirth self-efficacy. On their own, these findings might suggest limited impact. However, qualitative accounts told a more complex story. Many women described feeling more confident, more active, and more able to negotiate, adapt, and use movement during labor.

Taken together, these findings suggest that agency and self-efficacy may be temporally and contextually situated constructs. In nulliparous women, antenatal self-report measures are likely to capture anticipated confidence or intended agency, whereas labor is the setting in which these capacities are tested, negotiated, and made visible in practice. This interpretation is consistent with literature suggesting that empowerment-related constructs consolidate through lived experience and interaction with real care environments rather than through anticipation alone^32^.

The explanatory model developed in this study helps clarify this distinction. It suggests that decisional satisfaction and childbirth beliefs may be understood as proximal antenatal outcomes, whereas agency and self-efficacy become more visible as experiential intrapartum phenomena. This interpretation also helps explain why quantitative antenatal measures did not fully reflect changes that women later articulated in their postpartum narratives.

Mobility during labor emerged as the clearest behavioral link between antenatal preparation and childbirth experience. Women who reported remaining mobile during labor described more positive experiences overall, particularly in domains related to participation, own performance, and professional support. Qualitative accounts reinforced this pattern by portraying movement as a source of comfort, bodily control, confidence, and meaningful engagement with labor. These findings are consistent with evidence showing that movement and upright positions may support both physiological labor progress and women’s subjective experience of autonomy and control^2,4,8^. In the present study, mobility appeared to function not only as a clinical behavior, but also as a practical expression of agency. When women were able to move, they also appeared more likely to describe themselves as participating, coping, and aligning their bodily sensations with their preferences and interactions with professionals.

More broadly, the reduction in mobility after transfer to the birth room points to persistent institutional constraints, including routines and practices that may limit the enactment of previously prepared choices^33-36^. This reinforces the view that intervention effects were conditional rather than automatic.

Across both datasets, context emerged not as a background feature, but as a core condition shaping whether antenatal preparation could be translated into action. Women described situations in which institutional routines, clinical instructions, or limited flexibility constrained their ability to use movement or to act on their preferences. Nurse-midwives also identified contextual barriers, including limited space, large groups, and varying levels of professional comfort with participatory decision-support approaches. These findings suggest that individual capacitation, although necessary, is insufficient on its own. Agency during labor depended on whether the care environment allowed women to use what they had prepared, whether relational communication was supportive, and whether organizational conditions aligned with the intervention’s assumptions. This interpretation is consistent with literature describing the ways in which institutional routines can undermine women’s autonomy even when knowledge and motivation are present^10,33^.

The findings therefore point to an important implication: antenatal interventions designed to support agency may have limited reach unless they are accompanied by greater continuity and coherence between antenatal preparation and intrapartum care practices.

### Implications for practice and future research

The study supports the acceptability and feasibility of embedding experiential and decision-focused approaches within community-based childbirth preparation programs led by nurse-midwives. It also suggests several directions for intervention refinement, including strengthening the intensity of preparation, tailoring decision-support content to women’s needs, and improving continuity between antenatal and hospital-based care.

From a conceptual perspective, the study contributes by distinguishing between preparedness for action and agency enacted in labor, and by positioning mobility as a meaningful bridge between antenatal learning and childbirth experience. These insights may be useful not only for intervention refinement but also for future evaluation studies seeking to measure change more appropriately across pregnancy and childbirth.

Future research should build on these preliminary findings using adequately powered designs, ideally with comparison groups and longitudinal follow-up. Such studies should not focus exclusively on women’s individual preparation but should also address the organizational and professional conditions that shape whether agency can be enacted in labor. This may include engaging hospital-based teams, examining continuity across settings, and testing strategies that support both individual and system-level change.

### Strengths and limitations

This study has several strengths. First, the mixed-methods design enabled a more nuanced understanding of the intervention than either quantitative or qualitative data alone could provide. In particular, the integration of strands made it possible to explain why some constructs showed limited quantitative change despite being strongly expressed in women’s postpartum accounts. Second, the inclusion of both women’s and nurse-midwives’ perspectives allowed the analysis to address not only perceived impact but also implementation processes and contextual constraints. Third, the study was embedded within a feasibility framework informed by MRC guidance for complex interventions, which strengthened its developmental and interpretive coherence.

Several limitations should also be acknowledged. The small sample size limited statistical power, particularly for subgroup and multivariable analyses, and the absence of a control group precludes causal inference. Quantitative findings should therefore be interpreted as preliminary and explanatory. The sample consisted only of low-risk nulliparous women, most of whom had relatively high level of education, which may limit transferability to more diverse populations. In addition, women gave birth in different hospital settings, where practices relating to mobility, monitoring, and epidural use were variable, introducing contextual heterogeneity that could not be controlled. Finally, the researcher’s close involvement in the intervention may have supported implementation fidelity, but it may also have increased the likelihood of socially desirable responses in interviews.

Overall, despite these limitations, the study provides useful preliminary evidence that a woman-centered antenatal intervention may help strengthen women’s preparedness to act in relation to mobility during labor. At the same time, the findings indicate that the enactment of agency depends on whether intrapartum care environments are able to support, rather than constrain, women’s attempts to translate preparation into action.

## CONCLUSIONS

This mixed-methods study suggests that a woman-centered antenatal intervention focused on mobility during labor was acceptable and feasible for both women and nurse-midwives and may support women’s preparedness for action in childbirth. By integrating quantitative and qualitative findings, the study indicates that the intervention operated through cognitive, embodied, and relational mechanisms, moving beyond a purely informational model of antenatal education. The integrated findings suggest that the intervention’s most visible antenatal effects were improved satisfaction with decision-making and a stronger physiological orientation to childbirth, while agency became more clearly visible when women attempted to enact movement, participation, and decision-making during labor. Mobility appeared to function as an important bridge between antenatal preparation and childbirth experience, particularly in domains sensitive to agency, such as participation, perceived performance, and professional support. At the same time, the study highlights that preparation alone is not enough. Women’s ability to translate antenatal preparation into enacted agency depended on the relational and organizational conditions of intrapartum care. These findings underline the importance of aligning woman-centered antenatal interventions with care environments that support mobility, participation, and shared decision-making during labor.

Taken together, the findings contribute to understanding how antenatal midwifery interventions may support women not only to know more but to be better prepared to act. Future research should test these preliminary insights in larger and more contextually integrated studies that address both individual preparation and the care systems in which childbirth takes place.

## Data Availability

The data supporting this research are available from the authors on reasonable request. The data are not publicly available due to ethical and privacy restrictions.
